# Cross-linguistic regularities and learner biases reflect “core” mechanics

**DOI:** 10.1371/journal.pone.0184132

**Published:** 2018-01-11

**Authors:** Brent Strickland, Emmanuel Chemla

**Affiliations:** 1 Département d'Etudes Cognitives, Ecole Normale Supérieure, PSL Research University, Paris, France; 2 Institut Jean Nicod (ENS, EHESS, CNRS), Paris, France; 3 Laboratoire de Sciences Cognitives et Psycholinguistique (ENS, EHESS, CNRS), Paris, France; Northeastern University, UNITED STATES

## Abstract

Recent research in infant cognition and adult vision suggests that the mechanical object relationships may be more salient and naturally attention grabbing than similar but non-mechanical relationships. Here we examine two novel sources of evidence from language related to this hypothesis. In Experiments 1 and 2, we show that adults preferentially infer that the meaning of a novel preposition refers to a mechanical as opposed to a non-mechanical relationship. Experiments 3 and 4 examine cross-linguistic adpositions obtained on a large scale from machines or from experts, respectively. While these methods differ in the ease of data collection relative to the reliability of the data, their results converge: we find that across a range of diverse and historically unrelated languages, adpositions (such as prepositions) referring to the mechanical relationships of containment (e.g “in”) and support (e.g. “on”) are systematically shorter than closely matched but not mechanical words such as “behind,” “beside,” “above,” “over,” “out,” and “off.” These results first suggest that languages regularly contain traces of core knowledge representations and that cross-linguistic regularities can therefore be a useful and easily accessible form of information that bears on the foundations of non-linguistic thought.

## Introduction

Psychologists interested in the roots of human conceptual development have postulated the existence of a handful of “core” knowledge systems which could help get learning off the ground in infancy by orienting attention to important ontological categories (e.g. animate actors; physical objects) as well as by representing the principles guiding the behavior of the members of these categories [1]. Such core knowledge does not disappear once we exit infancy, but continues to structure mechanisms of language use [2–3] and visual perception [4] throughout the lifespan.

The current paper concentrates on a specific core system that has been postulated by psychologists: that of “contact mechanics” [5–6] which may form the foundations for learning about physical interactions between objects as well as artifact functions. We first provide theoretical evidence that some features of mechanical relationships (e.g. that of containment) are naturally more attention grabbing and conceptually salient than matched features of perceptually similar but non-mechanical relationships (e.g. that of occlusion). We then use these observations to motivate four empirical studies that ask whether traces of such attentional and conceptual prioritizations can be found in cross-linguistic regularities.

### Core mechanics?

A large body of work has shown that pre-verbal infants come to rapidly possess a great deal of knowledge about the physical world. One type of knowledge concerns the principles that govern the behavior of individual objects such as the principles of continuity [7] and cohesion [4]. A second form of knowledge concerns categories of contact mechanical interactions between physical objects such as causal launching [8], support [9], and containment [10].

To illustrate the relevant sense of contact mechanics here, consider the difference between containment and occlusion. Imagine that a ball is placed in a cup. If the cup moves, then so does the ball. Thus the movement of the cup contingently determines the movement of the ball. For the purposes of the current paper, we label this type of relationship as “mechanical” (the more formal way of defining a “mechanical” relationship is by saying that the relationship between objects A and B is mechanical if and only if, thanks to contact, the movement of one of the two objects can contingently determine the movement of the other object). On the other hand, if one places the ball *behind* the cup, then if one moves the cup, this most likely will not affect the location of the ball (unless one creates a mechanical relationship by bringing the two into contact). Given that the movement of the cup does not contingently determine the movement of the ball through contact, we label this relationship as being “non-mechanical.”

One emerging view from studies on non-verbal populations such as primates or pre-verbal infants as well as studies looking at adult visual perception, is that there may be a specialized mental system for representing mechanical relationships between objects [5,6]. In such a system, aspects of at least some mechanical (or potentially mechanical) relationships may be more attention grabbing and there representations more salient (i.e. more likely to be activated in thought) than perceptually similar but non-mechanical relationships.

A few theoretical and empirical considerations lend credibility to this view. First, there is now a substantial body of evidence suggesting that pre-verbal infants acquire knowledge about and show specific ways of attending to a range of contact-mechanical categories from early in development. Thus, research has revealed that by 4.5 months of age, infants understand the mechanical relationship of support, i.e., that unsupported objects fall [9], containment, i.e., that contained objects move when the container moves [11], and object solidity, i.e., that one solid object cannot pass through another [10]. Such abilities suggest that infants may preferentially attend to mechanical object relationships, which then leads to early learning.

This view is consistent with further recent evidence from [12] showing that from 6 months of age, infants will direct their attention to anticipated points of contact between the human body and functional artifacts. So for example, when an infant sees an adult pick up a phone, they will direct their attention to the person’s ear in anticipation of future contact between the phone and that point on the body, while when they see an adult pick up a (hair) brush, they will direct their attention towards the person’ hair, again in anticipation of contact. In these experiments, it is difficult to know if the infants’ attention to points of contact between the artifact and body is driven by attention to functional locations (which may be independent of contact locations) or attention to anticipated points of contact per se. Nevertheless, in the real world these properties are highly correlated, and the possible cognitive mechanisms not necessarily mutually exclusive. It may be that young infants spontaneously attend to contact object relationships, which in turn serves to support learning about artifact functions, and/or it may also be that they spontaneously attend to functional object parts. In either case, this would lead to widespread spontaneous attention to points of object contact or anticipated contact.

Theoretically, such a proposal of increased attention to specific types of domain relevant information is also in line with what we know about other core systems. Thus for example, pre-verbal infants distinguish animate from inanimate entities, and thereby preferentially attend to faces [13] and biological motion [14] over perceptually similar but inanimate static forms or motion types. This preferential attention may support learning about social agents [15] given that we are more likely to learn about those entities to which we attend. The implication here is that core mechanics may operate in a fashion analogous to the “core agency” system.

Attentional reflexes from core systems in development do not simply disappear when we grow up, but instead continue to exert an influence over the automatic allocation of attention even as adults. Thus, for example, adults, like infants, spontaneously attend more to animate than inanimate entities [16]. Continuing with the analogy, it may be the case that core mechanics operates in a similar fashion, with certain types of mechanical relationships attracting attention in adulthood in ways that mirror those found in infancy.

Some recent evidence suggests that this is indeed the case. Consider the contrast between containment and occlusion. Experiments employing a violation of expectation paradigm reveal that infants spontaneously encode information about object width at an earlier age than object height for containment events (4 months vs. 7.5 months [10,17]), while they encode object width and height at roughly equally ages for occlusion events (4.5 months vs. 4 months;[10,17]).

One interpretation of these data is that in containment events, pre-verbal infants preferentially attend to mechanically relevant information, since an object’s width, but not its height, predicts whether it will come into contact with the open mouth of the container or will fully pass inside.

When the visual system is put under load, adult attentional priorities for containment vs. occlusion events parallel those found in pre-verbal infants. In [18] the authors had adult participants view dynamic displays in which simple rectangles moved along vertical trajectories, while continuously disappearing and reappearing behind/in occluders/containers. Occasionally a randomly selected moving rectangle changed either its width or height, and the task was to detect these subtle changes in form. Mirroring the infancy results, adults were better at detecting width changes than height changes in containment events, while they were equally good at detecting width and height changes in occlusion events. This pattern of results held despite the fact that the majority of observers failed to report ever noticing the containment/occlusion difference in the displays, thus strengthening the conclusion that this pattern of results reflects automatic processes of visual attention as opposed to explicit response strategies.

In order to test whether these results truly reflected a process in which mechanically relevant features receive a higher priority in visual attention, subsequent experiments examined scenarios in which the static containers were lying on their sides so that rectangles now moved along *horizontal* paths in/behind and then back out. Consistent with the “mechanical” interpretation, height changes were detected more readily for containment events than width changes (since height now predicted whether the moving rectangles would fit inside the container or come into contact with the rim of the mouth), and there was still no difference in height vs. width accuracy for occlusion events.

Thus these results as well as those from the infancy literature are compatible with the view that mechanically relevant aspects of certain types of events and object relationships are naturally prioritized in visual attention (over non-mechanically relevant properties of those same event types).

In addition to the work on prioritized attention to mechanically relevant features of events, additional work with young infants suggests that mechanical relationships may be more salient than non-mechanical relationships. Here we are careful to distinguish between the highly related notions of conceptual salience and attention. Something X is prioritized over Y in visual attention if, all things being equal, X tends to spontaneously be attended to more frequently than Y. In our parlance, a representation of X is more conceptually salient than a representation of Y if the representation of X is more likely to be active in thought than the representation of Y. Attention is one way to generate conceptual salience since we are more likely to think about the things to which perceptually attend. However, conceptual salience can also apply whenever X and Y are not present and perceptible. For example, when considering a range of alternatives (e.g. places to eat) some might be more likely to come to mind than others even if none of the alternatives are within perceptual range and all the alternatives are plausible choices.

Returning to the topic of interest, there is independent evidence that infants treat contact mechanical relationships as more conceptually salient than non-mechanical relationships. So when 15–18 month old infants are asked to place two objects into a spatial relationship (with instructions using a spatial terms they do not understand), they first prefer to place the objects into a containment relationship. If no container is available, they then try to create a support relationship, and finally if neither containment nor support is possible, they prefer to place the two objects in contact [19–21]. They do not, however, show a systematic preference to place the object off of, beside, behind, or in front of the other object however. So despite that nothing in the task itself incentivizes creating contact mechanical relationships between objects, infants nevertheless spontaneously do this. This may be taken as evidence that such relationships are more conceptually salient than their non-mechanical alternatives.

### The relevance of language

Quite generally, language can be a useful “reflection” of how non-verbal knowledge is structured [22], and thus potentially provides a window into cognition about the physical world [23, 24]. In particular, regularities regarding language use and structure, including cross-linguistic regularities, may be revealing with regards to core elements of non-verbal cognition.

For example, many early emerging and foundational aspects of infant cognition (e.g. understanding the object vs. substance distinction; [25,26]), have close analogs in natural language syntax syntactic categories which regularly occur across languages (e.g. as in the mass/count distinction; [3]). Similarly, regularities in the frequency of use of number words across languages reflect basic aspects of number representation that are operational at an automatic level in adults as well as in pre-verbal infants [2].

The current paper examines the relationship between adpositional structure across languages and potentially “core” cognitive biases. More specifically if core cognition naturally prioritizes mechanically relevant object features in attention and makes mechanical object relationships conceptually salient (as defined above), then (1) adults should be more likely to ascribe the meaning of a novel preposition to a mechanical over a non-mechanical preposition since these meanings would be more salient and (2) words (and specifically adpositions) referring to mechanical relationships should more frequent and therefore systematically shorter across spoken languages.

This latter prediction follows from one prominent explanation for “Zipf’s law” [27] in natural language, whereby more frequently occurring words tend to be shorter. This law is robust across languages and syntactic categories [28], and has been posited to result from a trade-off between speakers’ and listeners’ effort [27,29–31]. One version of this idea is that once a word becomes frequent enough, the word is easier for listeners to identify, thus allowing energy saving speakers to drop some of its phonological elements (for empirical evidence of this phenomenon of phonological reduction see also [32]). In diachronic change, this ultimately leads to a shortening of the word. Here we hypothesize that increased conceptual salience for mechanical object relationships will in turn lead to increased frequency of usage for terms referring to such relationships, thus making them shorter.

While this connection between core cognition and word length has never been tested directly to our knowledge, the existing literature on (mostly English) prepositions is suggestive that biases generated by non-linguistic core cognition could serve to influence prepositional structure in the manner hypothesized here (we focus in this paper on prepositions and ad-positions because (1) these have received a large amount of attention in the linguistics literature and (2) because the majority of the data we received regarded adpositions. In principle however these considerations could apply to other syntactic categories).

First, it is commonly assumed that prepositions denote relationships that are represented by non-verbal aspects of cognition (e.g. [33]). Thus systematic differences in how certain object relationships are represented non-verbally could very well influence how those relationships are likely to be expressed verbally.

Secondly and more specifically, research investigating the psychological representation of preposition meaning has shown that the (perhaps tacit) grasp of mechanical relationships between objects (referred to as “functional relationships” in this literature) influences preposition use [34–36]. Thus for example, when a ball is located at the same level as the rim of the opening of a vertically oriented container, participants are more likely to say that the ball is “in” the container when the ball is in a mechanical relationship to the container (e.g. if the ball is supported by other balls which are in the container) than if the ball is not in a mechanical relationship with the container (e.g. if the ball is supported by a string from above) (see [36, 37]. Such findings suggest that, at least for certain prepositions such as “in” and “on” [36] patterns of meaning and usage are influenced by information about mechanics.

Finally, the pre-existing literature has emphasized a special role for attention in mediating between mechanical information (which is a particular type of functional information) and geometric information as presented in presented in perception [38], and this putative role is broadly in line with our general theoretical position as well as specific hypothesis. This work shows first that people naturally attend to the functional locations of objects (i.e. those object parts that are likely to come into contact with other objects). Secondly, it suggests that preferential attention to functional object parts influences the speed at which people can apply a preposition in context. For example, if an object is depicted above a water jug, participants will be faster at judging a sentence like “the object is above the water jug” if it appears close to the handle (which is likely to come into contact with the hand) as opposed to other parts of the jug.

### The current study

To recap, based on previous literature our theoretical perspective assumes that (at least some) mechanical object relationships are naturally salient (as well as attention grabbing). Here we focus on a specific set of predictions with respect to language use and structure that emerges from this view. Specifically people should (1) be disposed to think that a novel term refers to a mechanical over non-mechanical relationship (Note that some work has shown that even apparently non-mechanical object relationships such as “above” or “over” carry a functional component embedded in their meanings [34]. Even if they do involve attention to functional features, such cases involve specific knowledge of object categories (e.g. toothbrush over toothpaste), and would be expected to be limited to a narrower range of situations than mechanical object relationships) and (2) adpositions referring to mechanical object relationships should be used more frequently than those referring to non-mechanical relationships, thereby making them shorter across languages (in accordance with Zipf’s law). We test these predictions directly below

Two commonalities are present across the experiments. First, we specifically examine the containment (“in”) vs. occlusion (“behind”) contrast as a primary and direct test of this hypothesis. This contrast forms a minimally matched pair in the sense that each preposition can refer to very similar arrangements of objects, with only minor perceptual differences between the two. By picking this pair with such a minimal contrast, we also follow the developmental [10,11] and perceptual literature [18].

Across the three experiments we also tested the mechanical relationship of support (“on”) vs. a perceptually similar but non-mechanical relationship of being “above” (the relevant notion of “perceptual similarity” being used refers to the specific stimulus items we test in Experiments 1,2, and 3, in which many perceptual features between the mechanical and non-mechanical conditions are matched). The reason for studying “on” vs. “above” here is that, analogously to occlusion and containment, an understanding of both support and being above appears to be early emerging in infancy [39,9]. Despite the fact that some theoreticians [40] have speculated that the meaning of the non-mechanical “above” may not be naturally as salient as some mechanical relationships, this question has not received direct empirical attention. Given that this is an issue of active discussion in the literature, we test variants of this contrast in our stimulus sets below, with the prediction that if this mechanical relationship (like containment) is prioritized, then a representation of the support relationship (corresponding to “on”) may be treated as more salient than a representation of the spatial relationship corresponding to the meaning of the term “above”.

## Experiment 1

Here we test the mechanical relationships of “in” and “on”, and ask if these are prioritized over non-mechanical relationships in a meaning induction task. We assume here that all other things being equal, participants will be likely to assign the most contextually salient available meaning to a novel word. If the mechanical relationships studied here have a prioritized status (i.e. are considered highly conceptually salient, perhaps due in part to increased attention to mechanically relevant object features), this would predict that upon hearing a novel preposition, word learners should be more likely to think that the preposition refers to the mechanical relationship than to a closely matched but non-mechanical relationship.

### Methods

#### Participants

100 on-line participants were requested through Amazon’s Mechanical Turk, and 99 responses were received. Participants were recruited for token payment. All participants provided digital informed consent.

#### Stimuli and procedure

The "Conseil d'évaluation éthique pour les recherches en santé" provided approval for this study. Participants first viewed an attention check question (asking them *not* to indicate their level of education in a series of multiple-choice questions and to instead write “I have read the instructions” in a comment box).

**Fig 1:**
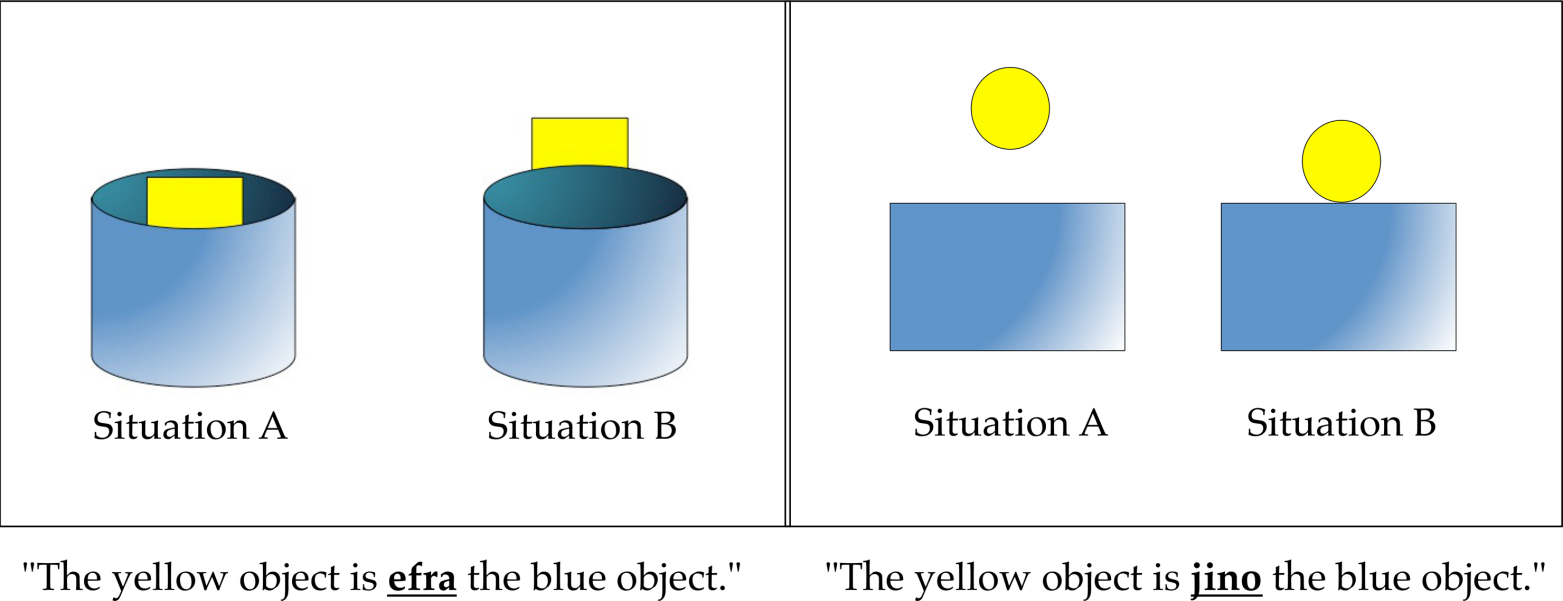
Depiction of the two test trials from Experiment 1. Participants’ instructions were as follows: “You hear the following sentence containing a preposition (in bold and underlined) from a foreign language. Which situation do you think this sentence refers to?”.

After the attention check, participants completed two test trials, presented sequentially in a randomized order. Each involved a single question. Test questions contained two pictures arranged side-by-side, one depicting a simple mechanical relationship between a yellow and a blue object (e.g. containment) and one depicting a closely matched but not mechanical relationship (e.g. occlusion). See Fig 1 for depictions of both trials. In each trial, the picture on the left hand side of the screen was labeled “Situation A” and the other “Situation B.” The left-right ordering of mechanical and non-mechanical pictures was balanced such that for one trial, the mechanical relationship was depicted on the left and for the other trial it was depicted on the right.

Below each picture, participants were presented with a sentence that they were told contained a preposition from a foreign language. The test sentences from each trial had a similar structure. Both prepositions were two syllables and four characters, and both prepositions were meaningless in English.

For example, in the containment scenario participants read, “the yellow object is **efra** the blue object.” The participants’ task was to indicate whether the preposition referred to Situation A (e.g., depicting the yellow object being in the blue object) or Situation B (e.g., depicting the yellow object being behind the blue object). Note that the non-mechanical relationships depicted here (“above” and “behind”) are referred to in English with two-syllable prepositions while the two corresponding mechanical relationships are referred to in English with one-syllable prepositions (“on” and “in”). Thus on the basis of syllable count, our stimuli were biased against our hypothesis in that they were actually more similar to the English prepositions which refer to the relevant non-mechanical relationships.

After responding to test questions, participants saw a series of demographic questions in which they provided their age, education level, native language, and any comments they may have had on the experiment.

### Results

11 participants who either failed the attention check or were not native English speakers were excluded prior to analyses. We thus analyzed the results of 88 native English speakers. We calculated each participant’s percentage of mechanical and non-mechanical responses across the test questions. Participants displayed a clear preference for mechanical over non-mechanical word meanings for the novel prepositions (65% vs. 35%). This difference was significant according to a Wilcoxon signed rank test (p < .002). This pattern of results held for both of the items tested. 54/88 participants chose the containment over occlusion while 60/88 chose support over above. Both of these were significant at the p < .05 level by a binomial test.

### Discussion

Participants displayed a clear preference to believe that a novel “foreign” preposition referred to the mechanical relationship instead of the non-mechanical relationship between objects. As Fig 1 makes clear, many of the low-level perceptual features in the contrasting “mechanical” and “non-mechanical” conditions are closely matched, making it unlikely that these would account for the effect. For example, all the object sizes were equated across the mechanical and non-mechanical conditions. Similarly, the alignment amongst objects along the horizontal axis was identical across conditions, so that the contained/supported/occluded/hovering objects were centered relative to the larger “ground” object (e.g. the container in containment events). The amount of visible surface area of the objects was also equated in the “on” vs. “above” item (though this was not controlled for in the containment vs. occlusion; an issue addressed in Experiment 2). The only low-level perceptual difference between the conditions in the “on” vs. “above” item was the positioning along the vertical axis between the figure and ground objects, which was necessary to implement the contact/no-contact distinction.

These results thus suggest that representations of mechanical relationships of containment and support are particularly salient in the context of inferring linguistic meaning.

## Experiment 2

**Fig 2:**
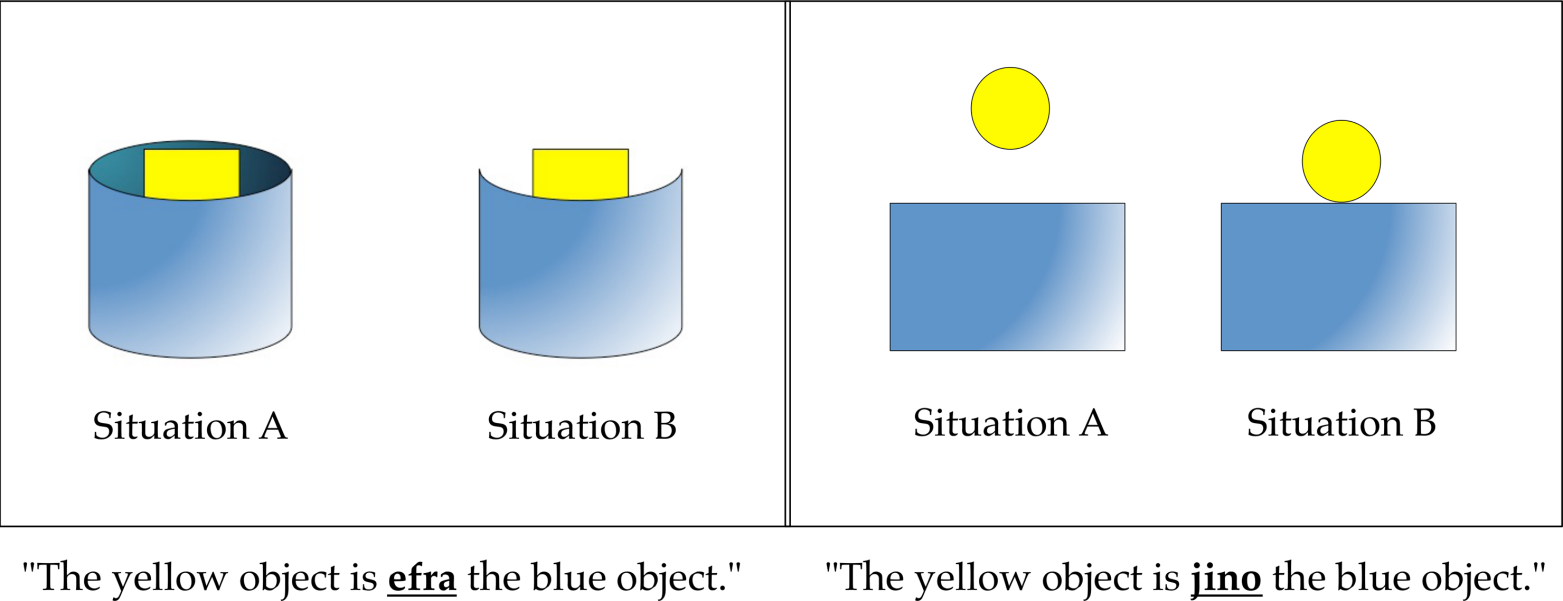
Depiction of test trials from the first block of Experiment 2.

Experiment 2 addressed two concerns with Experiment 1. First, in the depictions of the containment/occlusion contrast, the overall amount of visible surface area of the container/occluder differed between conditions, with more surface area being shown in the occlusion than the containment configuration. This confound was not present in the “on” vs. “above” scenarios, but it nevertheless remained a potential explanation for the preference to attribute the mechanical meaning to the foreign preposition in the containment/occlusion scenarios. In order to respond to this worry, here we reversed the visual pattern such that now more surface area was visible for containment than for occlusion configurations (see Fig 2). If the original preference for containment over occlusion could be explained by differences in visible surface area, here this preference should reverse.

Secondly, the overall pattern of results observed in Experiment 1 may be explained by a tendency to simply choose the depiction for which the likely corresponding English preposition is more frequent. For example, in the containment vs. occlusion scenarios, participants would likely describe these in English using “in” and “behind” respectively (even if these words never actually appeared as stimuli). On this view, since “in” is more frequent than “behind,” then participants would have a preference for the containment scenario. Similar concerns apply in the “on” vs. “above” contrast.

In order to deal with this second objection, we created two novel test items in which none of the depicted object relationships were mechanical. However, one scenario depicted a spatial relationship that would likely be described by a high frequency English preposition (e.g. “to”) while the other would likely be described by a lower frequency English preposition (e.g. “from”). To further ensure that participants were aware of the likely high vs. low frequency prepositions, we actually provided participants with the relevant terms. If the frequency explanation for the results of Experiment 1 were accurate, we should expect participants to systematically choose the high over low frequency meaning of the foreign preposition.

### Methods

#### Participants

100 on-line participants were requested and received through Amazon’s Mechanical Turk. Participants were recruited for token payment. All participants provided digital informed consent.

#### Stimuli and procedure

The stimuli and procedure for Experiment 2 were identical to those of Experiment 1 except in the following ways. For the depicted occlusion configurations, the backside of the container was removed so that in the containment scenario, there was more visible surface area showing than in the occlusion scenario.

**Fig 3:**
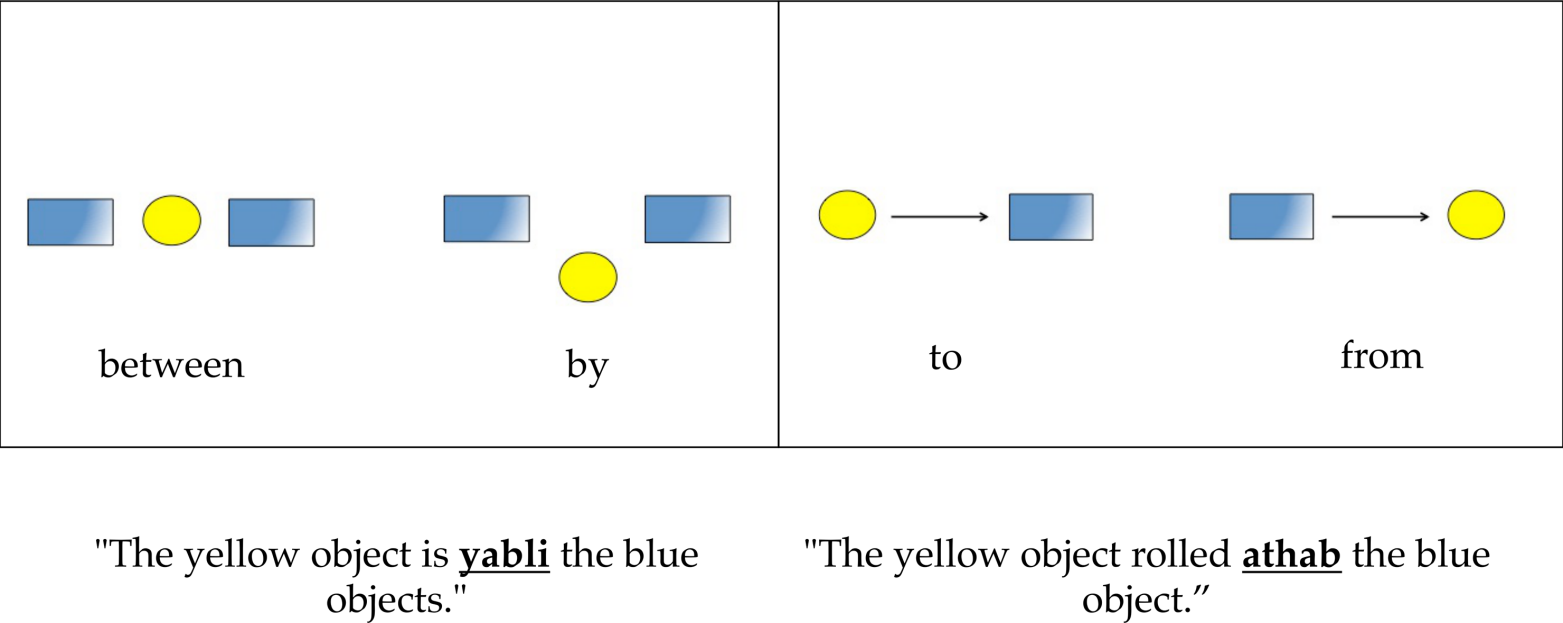
Depiction of test trials from the second block of Experiment 2.

Two new test trials were also created (see Fig 3). Each trial depicted two non-mechanical spatial relationships (between vs. by and to vs. from). One of the two spatial relationships was labeled with a high frequency term (“by”/“to”) while the other was labeled with an appropriate low frequency term (“between”/“from”). These trials were shown in a randomized order after a first block of trials including the containment/occlusion and “on” vs. “above” trials, thus allowing us to first replicate the effect found in Experiment 1 without interference from the new trial types.

Note that the differences in frequency between the high and low frequency items here were similar to the differences between the *likely* terms that people would use to refer to the spatial configurations in the test items from Experiment 1 (and the similar first block of items in Experiment 2). Thus according to the English Lexicon Project Website (http://elexicon.wustl.edu/; Balota et al., 2007) the HAL database raw frequencies for each relevant term were as follows: “in” (8,015,301) vs. “behind” (64,875); “on” (3,536,061) vs. “above” (177,919); “by” (1,790,440) vs. “between” (230,878); “to” (12,661,276) vs. “from” (1,894,943).

### Results

8 participants who either failed the attention check or were non-native English speakers were excluded prior to analyses. We thus analyzed the results of 92 native English speakers.

We first calculated each participant’s percentage of mechanical and non-mechanical responses across the test questions. These results almost perfectly replicated the results of Experiment 1. Participants again displayed a clear preference for mechanical over non-mechanical word meanings for the novel prepositions (66.3% vs. 33.7%). This difference was significant according to a Wilcoxon signed rank test (p < .001). This pattern of results held for both of the items tested. 63/92 participants chose the containment over occlusion while 59/92 chose support over above. Both of these were significant at the p < .05 level by a binomial test.

Secondly, we calculated each participant’s percentage of “high frequency” choices for the meaning of the foreign preposition in the second phase of the experiment. Participants actually displayed a preference for low-frequency terms for the novel prepositions (40.22% high frequency vs. 59.78% low frequency; p < .001). This significant difference was driven by the “by/between” trial. Thus for this item, 72/92 participants chose the *low* frequency choice (i.e. “between”), which was statistically significant (p < .001). For the “to/from” trial, participants showed no significant preference. Thus 54/92 chose the *high* frequency meaning (i.e. “to”), which was not statistically significant (p = .13).

### Discussion

These results replicate those from Experiment 1 showing that people display a tendency to assign mechanical over similar but non-mechanical meanings to novel prepositions (thus showing a tendency to believe that a novel preposition referred to containment and support object relationships over occlusion and “being above” respectively).

This experiment also addressed two concerns from Experiment 1. First these new results showed that the preference to think that a novel “foreign” preposition refers to a containment event over an occlusion event cannot be explained by the amount of overall surface area visible in depictions of the two configuration types. Secondly, the results from Experiment 2 ruled out the possibility that the observed preference to assign mechanical meanings to novel prepositions could have resulted from a preference to assign meanings associated with frequently used terms (such as “in” and “on”). Accordingly, participants failed to systematically assign the meanings of the frequently used terms “by” and “to” (instead of the infrequent “between” and “from”) to novel prepositions.

## Experiment 3

In Experiments 3–4 we ask whether the conceptual salience of mechanical relationships extends to production. Consistent with other recent work showing that word length is correlated systematically with factors relating to word meaning (Lewis & Frank, in press), we hypothesized here that words referring to mechanical object relationships may be systematically shorter than those referring to non-mechanical object relationships.

All things being equal, one would expect that people would be more likely to speak about situations in the world that are more salient to the observer. Thus if containment and support are more salient (due to increased attention, which may be a product of core cognition) in this way, one should expect that terms referring to these relations would be used more frequently. Due to Zipf’s law [27] the relevant mechanical terms should therefore be shorter across languages than those referring to similar but non-mechanical relationships.

We began by looking, across 38 languages at the length of terms referring to “in” and “on” vs. the closely matched but non-mechanical relationships of “behind,” “out,” “above,” “over,” “off”. In order to do this, we employed a novel method involving machine translation in which we translated each of the relevant prepositions into the 38 languages using an on-line website. Such a method has the major benefit of being fast and easy to use. The method may involve some degree of noise due to inaccuracies of machine translation, but over a large enough sample, one would expect this noise to even out to produce a reliable signal.

We again chose the test items based on the attention that the corresponding non-linguistic representations have received in the developmental literature, and due to the fact that this list only includes tightly controlled minimal contrasts.

### Methods

#### Stimuli and procedure

We translated prepositions from English into the following natural languages via the website nicetranslator.com (note that in some cases the translation of the English preposition is also a preposition in the second language, however this is not always the case. Experiment 3 addresses this issue in greater detail):

Arabic, Bulgarian, Catalan, Czech, Danish, Dutch, English, Estonian, Finnish, French, German, Greek, Haitian Creole, Hebrew, Hindi, Hmong Daw, Hungarian, Indonesian, Italian, Latvian, Lithuanian, Malay, Maltese, Norwegian, Persian, Polish, Portuguese, Romanian, Russian, Slovak, Slovenian, Spanish, Swedish, Thai, Turkish, Ukrainian, Urdu, Welsh.

The website allowed for the possibility of translation from English to languages with logographic writing systems as well as those with non-logographic writing systems. Given our interest in assessing word length, we studied only those languages with (non-logographic) segmental script writing systems, which offer a tight relationship between the length of a written word and its spoken length in these languages.

The range of languages was restricted by the website itself. We studied every possible naturally occurring language that had a non-logographic writing system and was made available by the website. We chose nicetranslator.com as a source of easy to extract information with a large database of languages, which for the purposes of the current experiment, could be seen as a random selection. Given that we had no prior knowledge of the website’s choice languages, and we sampled all non-logographic languages made available by the site at the time, we considered this method of (blind) sampling to be preferable to hand selecting languages since the latter strategy would leave open the possibility that we could hand select languages which were likely to be favorable to our hypothesis (see [41]).

The resulting 38 languages come from a variety of language families. A majority of 23 were Indo-European (9 Balto-Slavic; 6 Germanic; 6 Italic; 3 Indo-Iranian; 1 Hellenic; 1 Celtic); 3 were Afro-Asiatic; 2 Austronesian; 1 French Creole; 1 Hmong-Mien; 1 Tai-Kadai; 1 Turkic; 3 Uralic.

We concentrated on translations of the following two mechanical relationships (due to the prominence of their conceptual counterparts in the developmental literature): (1) “in” and (2) “on”. We examined them in contrast to minimally contrasting prepositions. “Behind” contrasts minimally (in terms of the spatial layouts described) with “in”; “out” is the antonym of “in” (according to many thesauruses though see [42] for evidence that people may not represent these as pure conceptual opposites); “above” and “over” both contrast minimally (in terms of spatial layouts) with “on”; “off” is the antonym of “on” (again according to many thesauruses though again see [42] for evidence that people may not represent these as pure conceptual opposites).

Each English word was entered into the online translator, which produced a translation for each of the 7 words in each of the 38 languages listed above.

### Results

The character counts of all the translated prepositions were calculated (spaces between letters were counted as a separate character), and then averaged to obtain an average length for mechanical and non-mechanical items within each language.

According to a Wilcoxon signed rank test the mechanical terms contained, on average, significantly fewer characters than non-mechanical terms (2.7 vs. 4.87, p < .001). This pattern (whereby mechanical contained fewer average characters than non-mechanical terms) held for 32/38 languages. We also ran a separate analysis looking only at the 13 non-Indo-European languages in our sample set. We analyzed things in this way because we were interested in assessing whether the expected pattern would hold over a diverse set of languages or whether the effects were driven primarily by the Indo-European languages.

In this sub-group, we again found a marginally lower number of characters for mechanical than non-mechanical terms (3.88 vs. 5.15, p = .08). This pattern held for 9/13 languages. We suspect that the fact that this difference was marginally significant, as opposed to fully significant, may have been due to a sample size problem in addition to the fact that the measure of the number of characters is somewhat noisy (Experiment 4 below addresses this problem by using a less noisy measure of word length and indeed finds a significant pattern of results for mechanical vs. non-mechanical items in non-Indo-European languages).

Item specific average character counts can be seen in Table 1. A Spearman’s correlation revealed a negative correlation with the frequency of use for the corresponding prepositions in English (rs = -.89, p < .01). This analysis suggests that, consistent with Zipf’s law, frequency of use causes the mechanical prepositions and their translations cross-linguistically to be systematically shorter.

**Table 1:** Row 1 lists average character lengths (and standard deviations) for translations of English prepositions across 38 languages, while row 2 lists word frequency. This is operationalized N-gram percentages (i.e. of all words present in a text, what percentage of those are the word in question) calculated for the year 2000 through a Google database of on-line books.

Measurement	"in"2	"on"	"behind"	"out"	"above"	"over"	"off"
Avg. Character count	2.92 (2.61)	2.71 (1.34)	5.87 (2.96)	4.24 (2.58)	6.47 (3.13)	4.66 (2.49)	4.45 (2.18)
English Ngram (year 2000)	1.56%	0.47%	0.01%	0.11%	0.02%	0.08%	0.03%

### Discussion

The current results suggest that the translations of the English mechanical prepositions “in” and “on” are systematically shorter than their non-mechanical counterparts (“behind,” “above,” “out,” “over,” “off”) across languages. Some readers may object that the use of character length as a measure of the length of spoken words is unreliable, but it is important to keep in mind that our analyses were limited to languages with (non-logographic) segmental script writing systems, which by definition closely map onto the phonological structure of the spoken language.

Thus the current results are consistent with the view that terms referring to the containment and support relationships are used more frequently, and therefore systematically shorter across languages.

## Experiment 4

The results of Experiment 3 were consistent with our guiding hypotheses. However, the use of machine translation introduced some methodological concerns. For example, the English prepositions tested here can be used in many different senses, and it may have been that the translator was not consistently providing translations for the relevant spatial sense(s) of the prepositions in question. Secondly, despite the correlational analyses presented above, one could still have doubts about using character count as a proxy for length.

In Experiment 4 language researchers from around the world were shown simple spatial depictions similar to those in Experiment 1 (e.g. a picture depicting a yellow object in a blue object), each accompanied by a sentence (e.g. “the yellow object is **in** the blue object”). The researchers provided translations of the English preposition into a language that they mastered, and provided an estimate of the syllable count. This methodology thus circumvents any potential problems with the on-line translation method used in Experiment 3.

### Methods

#### Participants

Researchers from the language sciences (linguistics, philosophy of language, psycholinguistics, etc.) with familiarity in a language that is not English were contacted on-line via personal emails, the parislinguists mailing list, and Linguistlist.org. We received 75 complete responses. Researchers translated into a total of 36 natural languages (we received multiple responses for some languages; languages are listed as reported by the researchers themselves; we excluded Esperanto from analysis because it is not a natural language). We analyzed the data from all the languages for which we received responses:

Arabic, Azerbaijani, Bosnian/Croatian, Boulou, Bulgarian, Burmese, Chinese, Czech, Danish, Dutch, Farsi, Finnish, French, Georgian, German, Hebrew, Hungarian, Italian, Japanese, Kannada, Lithuanian, Moroccan Arabic, Norwegian, Polish, Portuguese, Romanian, Russian, Sardinian, Serbian, Slovenian, Spanish, Swedish, Taiwan Mandarin, Turkish, Urdu, Vietnamese.

#### Stimuli and procedure

Researchers were invited to complete a short on-line questionnaire that worked in the following way. Researchers saw six total test trials. In each trial, they were shown a simple picture depicting either a mechanical (“in,” “on”) or non-mechanical relationship (“behind,” “beside,” “above,” “off”). The “in,” “on,” “behind,” and “above” pictures were identical to those depicted in Fig 1 (except that there was only one per trial). The “off” picture was also identical to the “above” picture from Fig 1, while the “beside” picture depicted a yellow rectangle just next to a blue container. Under the picture there was an English sentence describing the scenario (e.g. “The yellow object is **in** the blue object”). Participants were requested to translate the sentence into their chosen language, and put the translation of the preposition in parentheses. They were then asked to indicate the grammatical category of the translation (affix attached to verb, affix attached to noun, other type of affix, adposition, or other), the written form of the translation of the preposition, and finally, the syllable count of the translation of the specific word when spoken.

After the test phase, researchers also indicated their education level, field of study, age, level of mastery of English, and level of mastery of the language into which they were translating. Researchers additionally had the option of leaving open-ended comments or feedback if they so desired.

### Results

Expert respondents, on average, reported having a proficiency in English of 5.68 (on a scale of 1–7, with 7 being “native level proficiency”) while they reported having a proficiency of the non-English target language of 6.89, suggesting that the majority were native level or near native level speakers of the non-English language.

38 expert respondents reported having obtained a PhD. 28 reported having completed a master’s degree. 8 reported being a current graduate student (not having completed a master’s degree). 1 reported having only completed an undergraduate degree. 65 reported that their primary field of study was linguistics. 1 reported it to be philosophy, 3 psychology, 3 specializations in specific languages (e.g. French), and 3 reported having some other area of specialization related to the primary language sciences.

For each language, the reported syllable count of the translation for each mechanical and non-mechanical preposition was calculated. We then calculated the average mechanical and non-mechanical count (across languages). In some cases, participants mentioned that the translation could have varying numbers of syllables, depending on context. In these cases, we used the average of the number of syllables mentioned. A second nuance of the data is that upon receiving multiple responses for a given language, we used the average syllable count across individual responses to calculate a score for that specific language.

A related samples Wilcoxon signed rank test revealed that the mechanical words had systematically fewer syllables than the non-mechanical terms (1.64 vs. 2.38, p < .001). This pattern held for 31/36 languages. We also conducted an analysis which was restricted to the 15 non-Indo-European languages in our sample. This analysis again revealed a significant difference in length between mechanical and non-mechanical terms (2.09 vs. 2.62, p < .01). This pattern held for 13/15 languages. By-item results are listed in Table 2.

**Table 2:** Average character lengths (and standard deviations) for translations of English prepositions across 36 languages.

Measurment	"in"	"on"	"behind"	"beside"	"above"	"off"
Avg. syllable count	1.68 (.87)	1.6 (.92)	2.19 (1.04)	2.52 (.92)	2.26 (.95)	2.6 (1.3)

Finally, of the 214 data points we received (across languages), 167 of the terms were adpositions. We ran a restricted analysis that focused on those languages for which all of our respondents agreed that all the terms were adpositions. A related samples Wilcoxon signed rank test revealed that the mechanical adpositions had systematically fewer syllables than the non-mechanical adpositions (1.51 vs. 2.28, p < .001). This pattern held for 15 of the 16 languages in which we collected data for mechanical and non-mechanical adpositions.

### Discussion

The current results again suggest that the translations of the English mechanical prepositions “in” and “on” are systematically shorter than their non-mechanical counterparts (“behind,” “above,” “beside,” “off”) across languages.

## General discussion

The literature on infant and adult object perception had suggested that both populations spontaneously attend to mechanically relevant object features during ongoing events. From this we predicted that there would be an increased propensity to refer to relationships of containment and support rather than to occlusion and “being above,” thereby increasing the frequency of use of terms referring to mechanical relationships. In accordance with Zipf’s law, these more frequently used terms would thereby be systematically shorter.

Across four studies, we have found evidence in favor of our predictions. Native English speakers preferentially assign the mechanical meanings to novel prepositions. Moreover translations of “in” and “on” are systematically shorter than for matching non-mechanical terms such as “behind” and “above” across a wide range of languages, many of which are historically unrelated.

### The generality of the current effects

In the current paper, we have been careful to limit the scope of the conclusions about mechanical vs. non-mechanical relationships in core cognition given that our studies concentrate primarily on specific exemplars of the containment and support relationships (in addition to matching non-mechanical relationships) which are most likely to exemplify the core meanings of the corresponding prepositions (in English). We chose to concentrate on these specific relationships because of the attention that they have received in the developmental literature, and because, in the case of containment vs. occlusion, there were previously existing results that could be used to directly motivate a directional hypothesis.

The current results are consistent with two theoretical possibilities that are worthy of follow-up study, both of which would be compatible with the “core knowledge” framework. One possibility is that within the core system, all contact-mechanical relationships are more attention grabbing and conceptually salient than their non-mechanical counterparts. The second possibility is that the core system prioritizes functional features from only select range of event categories (e.g. causality, support, containment) but not others (e.g. attachment, covering). Follow-up experiments could investigate this issue employing visual search methods, infant looking methods, or methods like those employed here (though see below for some alternative explanations which are incompatible with the “core knowledge” framework).

### Prepositions and spatial cognition

There is a long-standing debate regarding the origins of the meanings of spatial adpositions. The “culture dependent” camp claims that linguistic meanings are built on highly culturally specific notions. On this view, one would expect large cultural variability in the range and type of spatial terms employed across languages [43–45]. On the other hand, “universalist” frameworks stipulate that spatial encoding systems should be highly related across languages since they are likely to draw from a common source of conceptual-spatial distinctions. On these views, cross-linguistic diversity should be minimal, and should stem from which particular subset of common conceptual distinctions a given language “chooses” to encode [46–48].

We interpret the current set of results as weighing in favor of the universalist views. Typologically recurrent patterns were observed across a diverse set of languages in the length of different types of spatial adpositions. These regularities were predicted from aspects of non-linguistic spatial representation observed in young infants and adult vision, and which are unlikely to be a product of culturally specific learning, at least according to most theories of “core knowledge” (e.g. [1]).

Our results and preferred interpretation would be compatible with a picture of the acquisition of spatial prepositions in which young children, when attempting to figure out the meanings of terms like “in,” “on,” “behind,” “off,” etc…, rely on a pre-existing and non-verbal ability to represent certain spatial configurations. Having this set of representations available scaffolds and supports the language acquisition process [19]. Thus the current results sit nicely with additional English data showing that the mechanical terms “in” and “on” are acquired earlier in language development than non-mechanical terms such as “under,” “behind,” and “off” [19,49].

It is important to point out that while our linguistic predictions were met, the current results do not conclusively show that mechanical object relationships are more attention grabbing or generally conceptually more salient (we instead assume this due to a specific reading of the background literature), nor do they directly show that such prioritizations, if they exist, are the cause of the linguistic patterns observed here.

Thus there is room for a “culture dependent” theorist to object to our preferred “universalist” interpretation. They may respond that while our results are indeed compatible with the view that mechanical relationships are referred to more often due to heightened conceptual salience, this could also be due to communicative or environmental factors as opposed to “core” ways of representing and attending to these object relationships non-verbally. For example, perhaps it is conversationally more informative to refer to specific types of mechanical than non-mechanical relationships.

This view however does not seem to make the correct predictions concerning the data. For example, a quick look around a cluttered desktop environment (such as that of the authors) reveals that support relationships may be at least as common as, if not more common than, “above” relationships. Thus supplying information about “above” relationships is likely to be either more informative (given the lower base rate) than supplying information about support or equally informative. Nevertheless, the conceptual representation of support (which is the meaning of “on”) seems to be more salient (Experiment 1,2) and “on” is systematically shorter (Experiment 3,4) than “above.” So the argument from informativity appears to make the wrong prediction.

Of course, considerations like these do not entirely rule out environmental or communicative factors as partial or full explanations for the effects observed here, but such views would need to be spelled out clearly and motivated by independent empirical evidence. For the moment, these elements are lacking, and we thus continue to believe that our explanation for the current results is the most plausible of the possible alternatives.

### New methods and future research

The current work could motivate follow-up studies which directly examine the relationship between spontaneous visual attention to mechanical relationships and frequency of reference. Specifically, our background assumption (based on our reading of the previous literature) was that mechanical relationships are naturally attention grabbing in vision. Tasks could be developed to more directly assess this (e.g. “spot containment amongst many partial occlusions” vs. “spot the partial occlusion amongst many containments”). Performance on such tasks could then be correlated with the propensity to refer to mechanical relationships.

Secondly, our findings are of relevance to developmental psychologists interested in the acquisition of spatial terms. As mentioned above, we suggest here that universal (non-verbal) “core” representations are responsible for the cross-linguistic verbal patterns observed. These same representations should be available to young children as they attempt to acquire the meanings of spatial terms (including adpositions) in the world’s languages. One would expect that the pattern cited in the above section (i.e. that “in” and “on” are acquired earlier in development than a wide range of non-mechanical terms) should be seen in development across the majority of the world’s languages. One particularly interesting question in any research that would look at this is whether advantages in children’s acquisition can be fully explained by the relative frequency of use of the various adpositions or whether there is an additional boost for the acquisition of mechanical terms, perhaps due to the conceptual salience of their meaning, that cannot be explained by word frequency alone.

Similarly, while some work on pre-verbal infants suggests spontaneous orientation of attention to anticipated points of contact [12] and prioritization for mechanically relevant object features during containment and occlusion [10, 17], these studies fail to show increased baseline looking times for containment vs. occlusion events, as might have been expected. This may be because infants and adults treat these events differently. Perhaps for example extensive exposure to different event types is necessary to bring about baseline differences in attention to each. However this may also be the case simply because the previous infant studies were designed to ask a different question (i.e. how infants will respond to apparent physical violations in each event type), and more sensitive measures such as preferential looking or eye-tracking would be needed to look at potential effects of increased attention to mechanical object relationships.

Finally, in addition to suggesting further research on both adults and infants on non-verbal object representation, the current paper also highlights the usefulness of a new and inexpensive tool: that of automatic machine translation. Machine translation provides an opportunity to obtain large amounts of data over a wide range of human languages in a cheap and quick way. It is true that this method has the down side of being less precise than structured data collection from human participants. However noise in the data due to a lack of precision can nevertheless be compensated for by large enough data sets (which this method allows one to obtain fairly easily). The reliability of the point can be appreciated by the fact that we obtained a very similar pattern of results in Experiments 3 and 4 despite the fact that Experiment 3 used machine translation and Experiment 4 involved collecting data from human participants.

We believe that this tool could be of use to researchers wishing to explore typological trends across large data sets, at least as a first step prior to running more structured data collection.

### Language and cognition

In summary, the current data suggest that a core knowledge property of conceptual salience may leave traces in cross-linguistic regularities, though alternative explanations remain possible. We proposed to look at two salient mechanical relationships encoded by the cognitive system from a very early age, and a fairly coarse and superficial linguistic measure: word length (or frequency). We submit that the simplicity of these measures make them potentially efficient and rich tools that should not be ignored in the investigation of the basic nature of certain cognitive abilities.

## Supporting information

S1 File Experiment 1. Includes all data for Experiment 1.
(XLS)

S2 File Experiment 2. Includes all data for Experiment 2.
(XLS)

S3 File Experiment 3. Includes all data for Experiment 3.
(XLSX)

S4 File Experiment 4. Includes all data for Experiment 4.
(XLSX)
